# Circular RNAs are differentially expressed in prostate cancer and are potentially associated with resistance to enzalutamide

**DOI:** 10.1038/s41598-019-47189-2

**Published:** 2019-07-24

**Authors:** John Greene, Anne-Marie Baird, Orla Casey, Lauren Brady, Gordon Blackshields, Marvin Lim, Odharnaith O’Brien, Steven G. Gray, Raymond McDermott, Stephen P. Finn

**Affiliations:** 1 Department of Histopathology and Morbid Anatomy, School of Medicine, Trinity College Dublin, Dublin 8, Ireland; 2 Department of Medical Oncology, Tallaght Hospital, Dublin 24, Ireland; 3 Thoracic Oncology Research Group, Trinity Translational Medical Institute, St. James’s Hospital, Dublin 8, Ireland; 4 Department of Clinical Medicine, Trinity College Dublin, Dublin 2, Ireland; 5 Cancer and Ageing Research Program, Institute of Health and Biomedical Innovation, Queensland University of Technology, Brisbane, Australia; 6 Department of Histopathology, St. James’s Hospital, Dublin 8, Ireland; 7 Labmed Directorate, St. James’s Hospital, Dublin 8, Ireland; 8 HOPE Directorate, St. James’s Hospital, Dublin 8, Ireland; 9 Department of Medical Oncology, St. Vincent’s Hospital, Dublin 4, Ireland

**Keywords:** Oncogenes, Prostate cancer

## Abstract

Most forms of castration-resistant prostate cancer (CRPC) are dependent on the androgen receptor (AR) for survival. While, enzalutamide provides a substantial survival benefit, it is not curative and many patients develop resistance to therapy. Although not yet fully understood, resistance can develop through a number of mechanisms, such as AR copy number gain, the generation of splice variants such as AR-V7 and mutations within the ligand binding domain (LBD) of the AR. circular RNAs (circRNAs) are a novel type of non-coding RNA, which can regulate the function of miRNA, and may play a key role in the development of drug resistance. circRNAs are highly resistant to degradation, are detectable in plasma and, therefore may serve a role as clinical biomarkers. In this study, AR-V7 expression was assessed in an isogenic model of enzalutamide resistance. The model consisted of age matched control cells and two sub-line clones displaying varied resistance to enzalutamide. circRNA profiling was performed on the panel using a high throughout microarray assay. Bioinformatic analysis identified a number of differentially expressed circRNAs and predicted five miRNA binding sites for each circRNA. miRNAs were stratified based on known associations with prostate cancer, and targets were validated using qPCR. Overall, circRNAs were more often down regulated in resistant cell lines compared with control (588 *vs*. 278). Of particular interest was hsa_circ_0004870, which was down-regulated in enzalutamide resistant cells (p ≤ 0.05, *vs*. sensitive cells), decreased in cells that highly express AR (p ≤ 0.01, *vs*. AR negative), and decreased in malignant cells (p ≤ 0.01, *vs*. benign). The associated parental gene was identified as *RBM39*, a member of the U2AF65 family of proteins. Both genes were down-regulated in resistant cells (p < 0.05, *vs*. sensitive cells). This is one of the first studies to profile and demonstrate discrete circRNA expression patterns in an enzalutamide resistant cell line model of prostate cancer. Our data suggests that hsa_circ_0004870, through *RBM39*, may play a critical role in the development of enzalutamide resistance in CRPC.

## Introduction

Prostate cancer (PCa) is the second leading cause of male cancer mortality in Western Europe and the United States^1^. Androgen deprivation therapy (ADT) is the mainstay of treatment^1^, with an average initial response of approximately 18 months, however resistance to ADT inevitably develops. This leads to castration-resistant prostate cancer (CRPC), which is currently incurable^2^. Although resistant to ADT, CRPC continues to rely on androgens via androgen receptor (AR) signalling^3^.

Enzalutamide is a targeted AR inhibitor that competitively binds to the ligand-binding domain (LBD) of the AR^4^. It inhibits AR translocation, recruitment of AR cofactors, and AR binding to DNA^4^. In previous phase 3 studies, enzalutamide prolonged overall and progression-free survival in patients who were chemotherapy naïve^5^, and in those who had previously received chemotherapy^4^. As a result, therapy with second generation anti-androgens has become recognised as a standard of care for advanced PCa^4,5^. Nevertheless, approximately 20 to 40% of patients will present with intrinsic resistance to enzalutamide as determined by sustained elevated prostate-specific antigen (PSA) levels and radiological or clinical progression^6^. Furthermore, patients who have an initial objective response will eventually develop secondary resistance^6^. While the exact mechanisms of enzalutamide resistance are yet to be fully understood, it appears that AR gene amplification emerges during treatment with ADT and facilitates tumour growth in low androgen concentrations^7^. Additionally, expression of the AR splice variant-7 (AR-V7), which is a truncated form of the AR lacking the ligand-binding domain^8^, has been shown to be associated with resistance to enzalutamide^6,9–11^. A number of mutations have also been identified in the AR in patients who are resistant to enzalutamide, such as F876L and may contribute to resistance^12,13^.

With the advances in experimental technology and bioinformatics, our understanding of RNA families has improved, as well as our general understanding of the importance of RNA associated interactions and subcellular locations^14,15^. One type of RNA family is non-coding RNA (ncRNA). ncRNA comprises of several different classes, including microRNAs (miRNAs) and long non-coding RNAs (lncRNAs), both of which are areas of active investigation in PCa^16^. A recently discovered novel ncRNA, called circular RNA (circRNA), may play an important role in cancer initiation, development, and progression^17–19^. circRNAs are RNA molecules with covalently joined 3′- and 5′- ends formed by back-splice events, thus presenting as closed continuous loops, which makes them highly stable^20,21^. They typically comprise of one to several coding exons of otherwise linear messenger RNAs (mRNAs) and range between a few hundreds and thousands of nucleotides in length^22^. Their high abundance, stability and evolutionary conservation between species suggest that they may have an important biological regulatory role^19^. circRNAs have been identified in a number of cancers including PCa^23^, suggesting a potential role as a biomarker or therapeutic target. Although, their role in cancer has yet to be fully elucidated, recent research suggests they can bind RNA-binding proteins (RBPs), translate peptides^24^ and confer resistance to therapy^25^. miRNAs have previously been shown to affect a wide array of biological processes and have an important role in regulating gene expression in cancer, where they act through downstream tumour-suppressive mRNAs^26^. It has been proposed that circRNAs can act as a miRNA ‘sponge’ thereby modifying miRNA activity through sequestration, thus altering mRNA target gene expression (34). circRNAs are extremely stable and resistant to RNA degradation, and as such they have the potential to translate into clinically useful blood based ‘liquid biopsies’ to detect early stage disease and monitor treatment response in real time. The goal of this study was to determine if circRNAs were differentially expressed in enzalutamide resistant cells, and to examine the circRNA-mRNA network involved in the development of drug resistance.

## Results and Discussion

### AR-V7 is elevated in enzalutamide resistant cells

**Figure 1:**
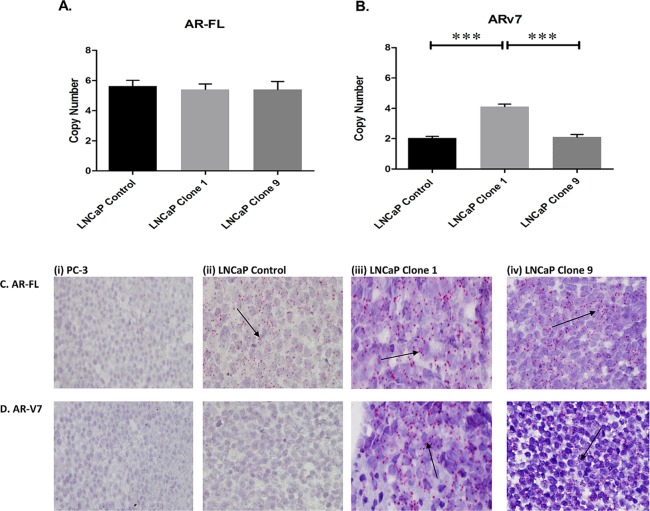
Expression of AR-FL and AR-V7 in an isogenic model of enzalutamide resistance. A standard curve method using a qPCR-based assay was used to determine the copy number of (**A**) AR-FL and (**B**) AR-V7. While AR-FL was consistent across all cell lines, AR-V7 varied according to enzalutamide resistance status. Data graphed as mean ± SEM (n = 3). Statistical analysis performed using ordinary one-way ANOVA (***p ≤ 0.001). RNA *in situ* hybridisation was used to determine the expression of (**C**) AR-FL and (**D**) AR-V7 in FFPE cell plugs. Positive expression was confirmed by the presence of punctate red staining (as indicated by arrow). No differences were observed for AR-FL, however AR-V7 expression was prominent in LNCaP clone 1 compared to the other two cell lines. PC-3 cells were used as a negative control. Representative images are shown at 40x magnification.

While it is known that the resistant cell lines used in this study harbour increased F876L, which is an agonist-switch mutation resulting in increased resistance to enzalutamide^12^, there is no information on it’s association with AR-V7 levels. We detected AR-FL and AR-V7 expression in the cell line model using a standard curve qPCR method. While, AR-FL copy number was consistent across the panel (Fig. 1A), AR-V7 copy number varied depending on enzalutamide resistance status (Fig. 1B). AR-V7 was significantly elevated in LNCaP clone 1 (highly resistant) compared with LNCaP control (sensitive) (p ≤ 0.001). AR-V7 was also higher in LNCaP clone 1 compared with LNCaP clone 9 (moderately resistant) (p ≤ 0.001). The expression of AR-FL (Fig. 1C) and AR-V7 (Fig. 1D) was confirmed using RNA *in situ* hybridisation (RISH) (BaseScope™). Qualitatively, AR-V7 expression varied across cell lines, with the highest expression in clone 1 and no expression detected in the control cell line (Fig. 1D). Enhanced levels of AR-V7 are associated with increased drug resistance^6,27^.

### circRNA screening identified differentially expressed profiles within an isogenic model of enzalutamide resistance

**Figure 2:**
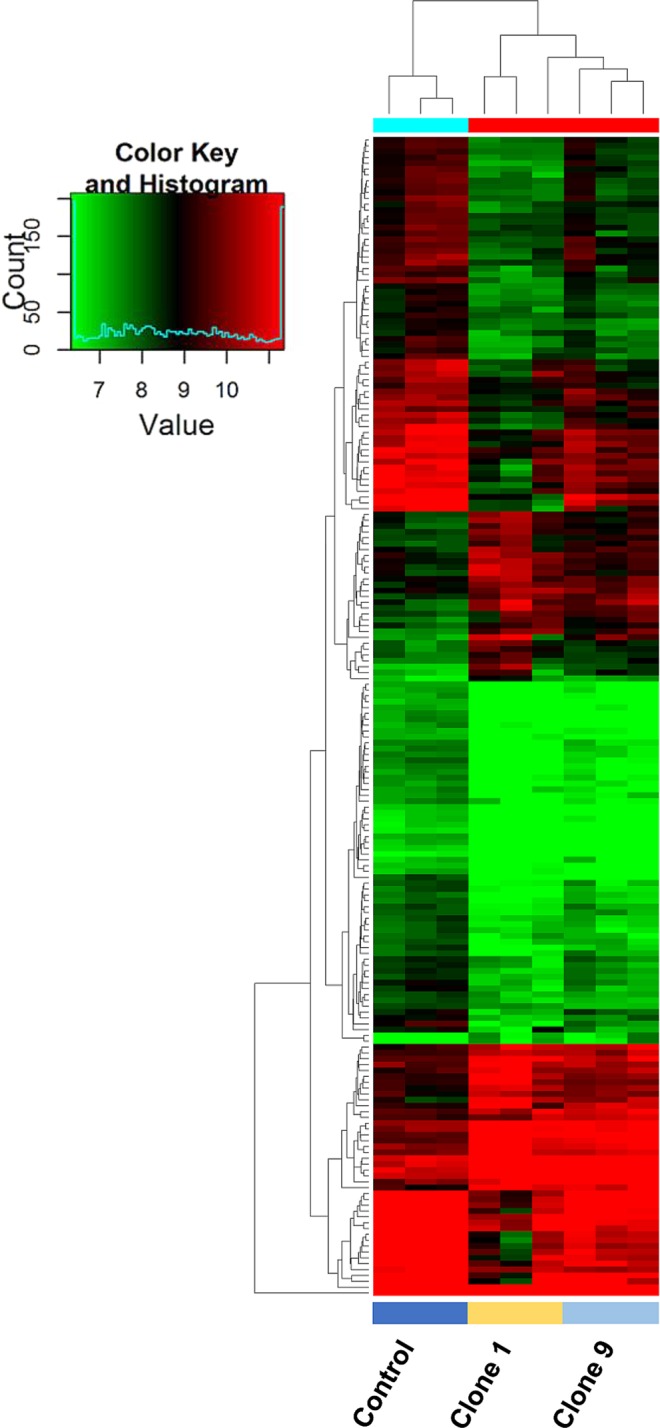
Heatmap demonstrating changes in circRNA expression in clone 1, and clone 9 *vs*. control. Unsupervised clustering (euclidean distance measure and the ‘average’ agglomeration method) was used for analysis (n = 3). Red indicates higher levels of expression, while green indicates lower levels of expression.

To determine differential expression of circRNAs within the dug sensitive (control) and resistant clones (clone 1 and clone 9), cell lines were screened for circRNA expression using a circRNA 2.0 microarray (Arraystar), which covers 13,617 circRNAs. In total, 930 circRNAs were classified as present across the panel of three cell lines. These target circRNAs were used for further differential analysis. The fold change (FC) for each circRNA between two groups (control *vs*. combined clone1/9) was computed. A student’s paired *t* test was then used to identify significantly altered circRNAs. The false discovery rate (FDR) was applied to determine the threshold of p value. circRNAs with FC ≥ 1.5 and p < 0.05 were considered to be significantly differentially expressed. Grouped analysis (control *vs*. combined clone1/9) of detected circRNAs according to FC was performed. Overall, more circRNAs were significantly down-regulated in the enzalutamide resistant cell lines compared with the control. There were 278 circRNAs significantly up-regulated (p < 0.05, control *vs*. combined clone1/9) and 588 circRNAs that were significantly down-regulated (p < 0.05, control *vs*. combined clone1/9). Data is presented as a heat map in Fig. 2. A complete list of circRNAs is accessible through the GEO Series accession number GSE118959 (https://www.ncbi.nlm.nih.gov/geo/query/acc.cgi?acc=GSE118959)^28^ and is provided in Supplementary Table 1.

### The circRNA profile is further altered depending on the extent of enzalutamide resistance

**Figure 3:**
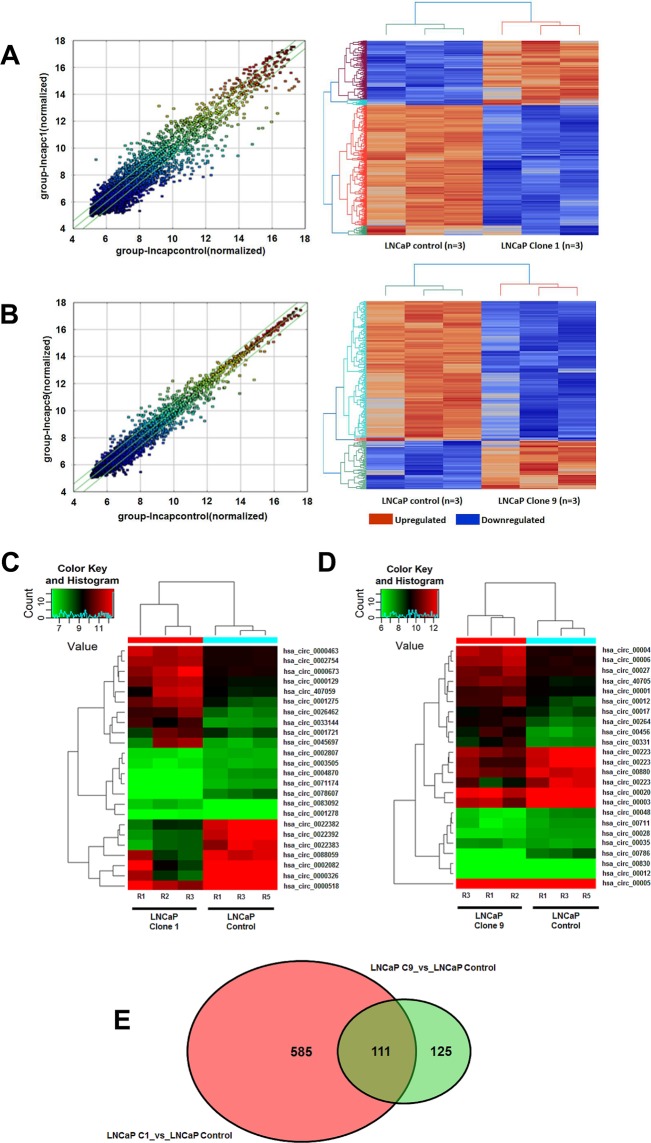
Scatterplot and matching heatmap of circRNA expression between (**A**) control and clone 1 and (**B**) control and clone 9. The values of X and Y axes in the scatterplot are the normalized signal values of the samples (log_2_ scaled) or the averaged normalized signal values of groups of samples (log_2_ scaled). The green lines in the scatterplot indicate FC. Heatmap reflects changes in expression using unsupervised clustering analysis (euclidean distance measure and the ‘average’ agglomeration method) (n = 3). Red indicates higher levels of expression, while blue indicates lower levels. circRNAs chosen for validation are outlined in smaller heat maps showing the top five up and down regulated circRNAs in clone 1 *vs*. control (**C**) and clone 9 *vs*. control (D)(n = 3). Green indicated reduced expression, with red indicating increased expression. (**E**) Venn diagram displaying differentially expressed and overlapping circRNAs between clone 1 and clone 9 *vs*. control.

Differential circRNA expression was also evident depending upon the extent of enzalutamide resistance (Fig. 3).

#### Clone 1 vs. control

In clone 1, we identified 230 up-regulated circRNAs (p < 0.05, *vs*. control), and 465 that were down-regulated (p < 0.05, *vs*. control). Thus, indicating the changing levels of circRNAs as enzalutamide resistance develops and levels of AR-V7 increases. Data is shown as a scatterplot and associated heatmap in Fig. 3A,C. A complete list of circRNAs is provided in Supplementary Table 2.

#### Clone 9 vs. control

In terms of clone 9, we discovered 60 up-regulated circRNAs (p < 0.05, *vs*. control), and 175 that were down-regulated (p < 0.05, *vs*. control). Data is shown as a scatterplot and associated heatmap in Fig. 3B,D. A complete list of circRNAs is provided in Supplementary Table 3. A Venn diagram is provided to show the overlap and different levels of expression between clone 1 and clone 9 with control (Fig. 3E). This Venn diagram display 585 circRNAs that were differentially expressed clone 1 *vs*. control but not clone 9 (shown in red) and 125 differentially expressed circRNAs between clone 9 *vs*. control but not clone 1 (shown in light green). There were 111 differentially expressed circRNAs common to both clone 1 and clone 9 *vs*. control (dark green).

Data is accessible through the GEO Series accession number GSE118959 (https://www.ncbi.nlm.nih.gov/geo/query/acc.cgi?acc=GSE118959).

### Associated circRNA parental genes are involved in pro-oncogenic activities

The top 5 circRNAs, ranked by FC, are shown in Table 1 for clone 1 *vs*. control; and in Table 2 for clone 9 *vs*. control. The parental genes of differentially expressed circRNAs were obtained from circBASE database (www.circbase.org)^29^. hsa_circ_0001275 was up-regulated in clone 1 *vs*. control (p = 0.047). The associated parental gene is *PLCL2*. Previously, *PLCL2* (Inactive phospholipase C-like protein 2) was identified as part of a 23-gene signature, which predicted metastatic-lethal PCa outcomes in men diagnosed with clinically localised PCa^30^. hsa_circ_0022392 was down-regulated clone 1 *vs*. control (p = 0.0002) and is associated with the gene *FADS2* (Fatty acid desaturase 2), which may have a role to play in cancer development^31^. In clone 9, hsa_circ_0045697 is up-regulated (p = 0.029, *vs*. control) and is associated with the oncogene *ITGB4* (Integrin Subunit Beta 4). Studies have shown that *ITGB4* promotes prostate tumourigenesis^32^. Further information is outlined in Tables 1 and 2.

**Table 1:** Top five up and down-regulated circRNAs in clone 1 *vs*. control based on FC.

CircRNA	Genomic Location	Expression	Fold Change	p-value	Parental Gene	Gene Function
hsa_circ_0001275	chr3:17059499-17059748	up	5.8	0.0473	*PLCL2*	Complimentary to Gleason score for the prognostic classification of patients PCa^30^
hsa_circ_0026462	chr12:53068519-53069224	up	5.7	0.0260	*KRT1*	Target receptor highly expressed on breast cancer cells^41^
hsa_circ_0033144	chr14:99723807-99724176	up	5.2	0.0128	*BCL11B*	Methylated in PCa^42^
hsa_circ_0000673	chr16:11940357-11940700	up	4.2	0.0383	*RSL1D1*	Overexpression is associated with an aggressive phenotype and poor prognosis in patients with PCa^43^
hsa_circ_0000129	chr1:151145974-151149507	up	3.9	0.0385	*VPS72*	May have a role in regulating long-term hematopoietic stem cell activity^44^
hsa_circ_0022392	chr11:61630443-61631258	down	20.2	0.0003	*FADS2*	Polymorphisms in the FADS gene cluster may have an impact on the effect of ω3 and ω6 PUFA on PCa risk amongst different populations^45^
hsa_circ_0022383	chr11:61605249-61615756	down	15.9	0.0011		
hsa_circ_0022382	chr11:61605249-61608197	down	14.6	0.0002		
hsa_circ_0000518	chr14:20811404-20811554	down	16.2	0.0281	*RPPH1*	ncRNA involved in processing of tRNA precursors by cleaving the trailer sequence from the 5′-end^46^
hsa_circ_0071174	chr4:151656409-151729550	down	2.4	0.0031	*LRBA*	LRBA has been implicated in regulating endosomal trafficking, particularly endocytosis of ligand-activated receptors^47^

**Table 2:** Top five up and down-regulated circRNAs in clone 9 *vs*. control based on FC.

CircRNA	Genomic Location	Expression	Fold Change	p-value	Parental Gene	Gene Function
hsa_circ_0045697	chr17:73736438-73753899	up	4.7	0.0297	*ITGB4*	Involved in prostate tumorigenesis and cancer invasiveness^32^
hsa_circ_0000463	chr12:132609079-132609271	up	4.0	0.0088	*EP400NL*	Pseudogene
hsa_circ_0026462	chr12:53068519-53069224	up	3.5	0.0254	*KRT1*	Target receptor highly expressed on breast cancer cells^41^
hsa_circ_0000673	chr16:11940357-11940700	up	3.5	0.0054	*RSL1D1*	Overexpressed in PCa^43^
hsa_circ_407059	intronic	up	3.2	0.0018	*FGFR1*	Role in prostate tumorigenesis (40)
hsa_circ_0000326	chr11:65272490-65272586	down	6.4	0.0351	*XLOC_l2_002352*	Undefined
hsa_circ_0022383	chr11:61605249-61615756	down	6.1	0.0244	*FADS2*	Polymorphisms in the FADS gene may have an impact on the effect of ω3 and ω6 PUFA on PCa risk among different populations^45^
hsa_circ_0022392	chr11:61630443-61631258	down	4.1	0.0298		
hsa_circ_0078607	chr6:160819010-160831878	down	5.9	0.0113	*SLC22A3*	Contributes to PCa pathogenesis^48^
hsa_circ_0002082	chr11:65271199-65272066	down	5.8	0.0371	*MALAT1*	Plays a role in AR-V7 resistance^49^

### miRNAs are associated with circRNAs

circRNAs contain multiple sites called miRNA response elements (MREs) which are miRNA binding sites found on circRNAs^18^. circRNAs can bind up to five different miRNAs. For this study, the miRNAs were predicted using Targetscan^33^ and miRanda^34^ bioinformatic platforms. This bioinformatics approach determined which probable miRNA was associated with each circRNA. For each identified circRNA, the top five most likely miRNA binding sites were predicted. The circRNAs were then filtered according to miRNAs that were strongly associated with PCa in the literature (Table 3), thus producing a list of ten relevant up-regulated and down-regulated circRNAs for validation (Table 4) (41). Further information relating to corresponding parental gene, MREs, miRNAs, and associated miRNA function is outlined in Table 4.

**Table 3:** miRNAs associated with circRNAs, with a known involvement in PCa.

**miRNA**
mir-141
mir-181a
let-7b
mir-125b
mir-145
mir-205
mir-221
mir-222
mir-25
mir-93
mir-21
mir-34a
mir-521
mir-106b
mir-96
mir-124
mir-449b
mir-23b
mir-124
mir-27b

**Table 4:** List of circRNAs selected for validation with their corresponding parental gene, MREs, miRNAs, and associated miRNA function.

CircRNA	Genomic Location	Expression	Fold Change	p-value	Parental Gene	MRE	Gene Function
hsa_circ_0004870	chr20:34302106-34313077	down	2.4	0.0015	*RBM39*	miR-145	Cancer cell migration and invasion^50^
hsa_circ_0002807	chr13:114149816-114164739	down	1.7	0.0009	*TMCO3*	miR-141	Suppresses stem cells^51^
hsa_circ_0022383	chr11:61605249-61615756	down	6.1	0.0244	*FADS2*	miR-124	Inhibits invasion and proliferation^52^
hsa_circ_0003505	chr17:20910208-20911309	down	1.6	0.0421	*USP22*	miR-124	Inhibits invasion and proliferation^52^
hsa_circ_0088059	chr9:114905750-114905903	down	2.5	0.0281	*SUSD1*	miR-124	Inhibits invasion and proliferation^52^
hsa_circ_0000673	chr16:11940357-11940700	up	3.5	0.0053	*RSL1D1*	miR-25	Modulates invasiveness and dissemination^53^
hsa_circ_0002754	chr8:41905895-41907225	up	2.3	0.0493	*KAT6A*	miR-145	Cancer cell migration and invasion^53–55^
hsa_circ_0001278	chr3:31617887-31621588	up	1.6	0.0004	*STT3B*	miR-205	ERG target gene^56^
hsa_circ_0001721	chr7:90355880-90356126	up	1.9	0.0103	*CDK14*	miR-221	Promotes cell proliferation and represses apoptosis^35^
hsa_circ_0083092	chr7:155471301-155473602	up	2.4	0.0051	*RBM33*	miR-125b	Tumour suppressor^57^

### Validation of circRNAs

**Figure 4:**
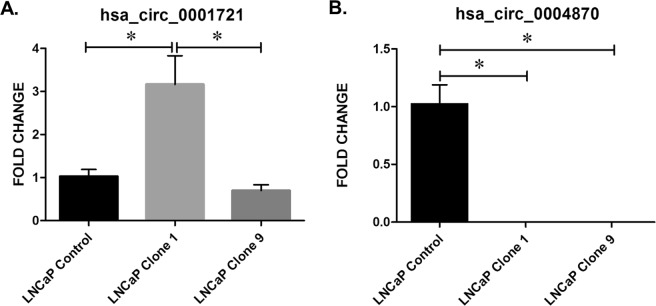
Validation of candidate circRNAs in an isogenic model of enzalutamide resistance. (**A**) hsa_circ_0001721 and (**B**) hsa_circ_0004870. Data graphed as mean ± SEM (n = 3). Ordinary one-way ANOVA (*p < 0.05).

Custom designed outward facing primers were designed for use with qPCR for selected circRNAs (Table 5). hsa_circ_0001721 was significantly up-regulated in clone 1 *vs*. control (p ≤ 0.05), which corresponded to the array data (Fig. 4A). Similarly, hsa_circ_0001721 was significantly up-regulated in the more resistant clone 1 *vs*. clone 9 (p ≤ 0.05) (Fig. 4A). hsa_circ_0001721 is an exonic circRNA, located on chromosome 7 and is associated with the gene *CDK14*^35,36^. hsa_circ_0004870 was significantly down-regulated in clones 1 and 9 *vs*. control (p ≤ 0.05) (Fig. 4B). hsa_circ_0004870 is an exonic circRNA located on chromosome 20 and is associated with the gene *RBM39*^37^.

**Table 5:** Primers used in this study.

circRNA	Primer Sequence (5′-3′)
000487	F: TGGGAACAACTGGTCGTCTT
	R: CTTGGTCGAATTCTTGCCGC
0001278	F: CGGTCAGTAGCTGGATCCTT
	R: ACCATGCTCTTTCATCAAACCA
0002807	F: TTCCACGTGTCTGTCCTTGT
	R: ACAGCAATCCACGGGTCTCT
0022383	CCACAAGGATCCCGATGTGAA
	TTCACCAATCAGCAGGGGTT
0003505	GCGGAAGATCACCACGTATG
	CAACCGCTGCACTTGATCTT
0000673	TGACTGTATAGGTGGAACAGTCT
	AAAACTGCTCAGAAGGCGGA
0002754	ACCAACGTGGATGGGAAAGA
	TCCCCAAGAAACTAGTCAGCAC
0001278	CGGTCAGTAGCTGGATCCTT
	ACCATGCTCTTTCATCAAACCA
0001721	TCCTCCACTGGCAAAGAGTC
	CAGGAATTGTGTCCAGGGGTT
0083092	CCAGAGGAGGAGCAGCTTTAC
	CCAGAGGAGGAGCAGCTTTAC

### hsa_circ_0004870 may have a role in splicing via U2AF65

**Figure 5:**
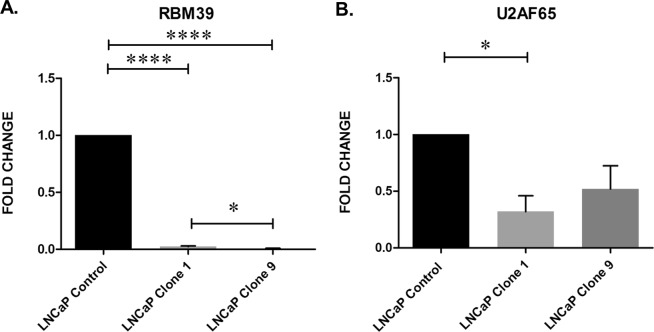
hsa_circ_0004870 expression according to (**A**) malignancy status and (**B**) and androgen dependency. Data graphed as mean ± SEM (n = 3). Ordinary one-way ANOVA (*p < 0.05, ****p ≤ 0.0001).

**Figure 6:**
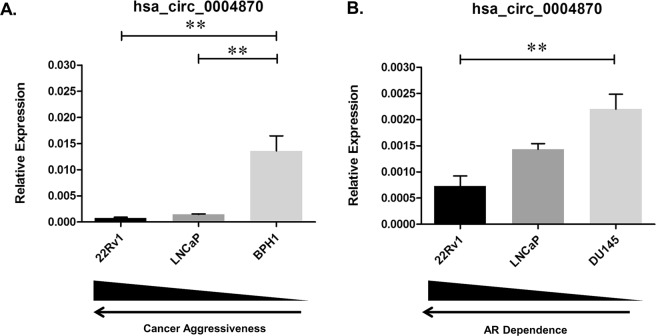
Expression of (**A**) RBM39 (**B**) and U2AF65 in the isogenic model of enzalutamide resistance. Data graphed as mean ± SEM (n = 3). Ordinary one-way ANOVA (**p < 0.01).

Previous studies have demonstrated that circRNAs are down-regulated in cancer^17^, therefore we selected hsa_circ_0004870 for further investigation. We confirmed that hsa_circ_0004870 was down-regulated in LNCaP (p ≤ 0.01) compared with the benign prostatic hyperplasia line, BPH1 (Fig. 5A). Similarly, hsa_circ_0004870 was down-regulated in the AR positive 22Rv1 cell line (p ≤ 0.01) compared with the AR independent line, DU145 (Fig. 5B). The coordinates for hsa_circ_0004870 (chr20:34,302,106-34,313,077), correspond to the gene *RBM39* on the UCSC Genome Browser, thus identifying this as the parental gene. RBM39 is a serine/arginine-rich RNA-binding protein thought to activate or inhibit the alternative splicing of specific mRNA by interacting with the spliceosomal components within splice sites^37^. *RBM39* was significantly down-regulated in the resistant clones 1 (p ≤ 0.0001) and clone 9 (p ≤ 0.0001) compared with control (Fig. 6A). *RBM39* encodes a member of the U2AF65 family of proteins and it has previously been shown that, U2AF65 leads to expression of AR-V7 via the lncRNA, PCGEM1, binding to AR pre-mRNA^38^. We confirmed expression of U2AF65 in the cell line panel, which was significantly down-regulated in the clone 1 (p ≤ 0.05) (Fig. 6B). Our data has shown that RBM39 and U2AF65 are down-regulated in clone 1, which has the highest expression of AR-V7. This may be due, in part, to high turnover of mRNA, or circRNA regulation of alternate pathways. This data suggests that the deregulation of hsa_circ_0004870 may be associated with the development of drug resistance through the regulation of AR-V7.

## Conclusion

circRNAs have been identified in a number of different cancers (90), suggesting a potential role as a biomarker or therapeutic target. Although, their role in cancer has yet to be fully elucidated, recent research suggests they can act as miRNA sponges (170), bind RNA-binding proteins (RBPs), translate peptides (83) and may confer resistance to therapy (192). In this study, we report for the first time, to the best of our knowledge, circRNA expression profiles associated with enzalutamide resistant PCa. Our findings indicate that circRNAs may potentially represent valuable prognostic and diagnostic biomarkers in the real time monitoring of treatment response to enzalutamide. Given that other studies have shown circRNAs to be abundant, highly stable, and detectable in human saliva, tissue and blood samples^22,39^, their potential as liquid based biopsy markers is evident, in addition to their capacity to serve as a predictive marker in this disease.

## Methods

### Cell lines

The isogenic enzalutamide resistance LNCaP model was gifted from Novartis^12^. The panel consisted of an aged match control cell line (drug sensitive), and two sub-lines termed clone 1 and clone 9. Clone 1 was most resistance to the drug, with clone 9 displaying moderate resistance. Cells were cultured in RPMI-1640 media (Merck KGaA, Darmstadt, Germany) with 10% FBS (Merck KGaA) and 1% Penicillin Streptomycin (Merck KGaA). PC-3 were cultured in ATCC-formulated F-12K Medium containing 10% FBS and 1% Penicillin Streptomycin (Thermo Fisher Scientific, CA, US).

### RNA preparation

Total RNA was prepared from cell lines from three independent experiments using TRIzol (Life Technologies, CA, USA) according to manufacturer’s instructions. Subsequently, the RNA underwent DNase treatment with Ambion® TURBO™ DNase (Thermo Fisher Scientific, MA, USA) and a further RNA clean-up was performed using standard ethanol precipitation protocol.

### circRNA microarray

Cell line (from three independent biological replicates) analysis was performed using the Arraystar Human circRNA Array version 2.0 (Arraystar, Rockville, MD, USA). The sample preparation and microarray hybridization were performed according to manufacturer’s instructions. Briefly, total RNA was digested with RNAse R (Epicentre, Illumina, San Diego, CA, USA) to remove linear RNAs and enrich for circRNAs. The enriched circRNAs were amplified and transcribed into fluorescent cRNA utilizing a random priming method Arraystar Super RNA Labelling Kit (Arraystar). The labelled cRNAs were hybridized onto the Arraystar Human circRNA Array V2 (8 × 15 K). The array slides were washed and scanned on the Agilent Scanner G2505C. Agilent Feature Extraction software (version 11.0.1.1) was used to analyse acquired array images.

### Microarray data analysis

Quantile normalization and subsequent data processing were executed using R software package^40^. CircRNAs with at least 4 out of 8 samples that were flagged as present or marginal (an attribute that denotes the quality of the entities) were considered to be target circRNAs according to GeneSpring software’s definitions and instructions. CircRNA and miRNA interactions were predicted with the Arraystar’s miRNA target prediction software based on TargetScan^33^ and miRanda^34^. These target circRNAs were used for further differential analysis.

### Quantitative real-time PCR

cDNA was synthesized from 1 µg RNA using a High Capacity cDNA Reverse Transcription Kit (Thermo Fisher Scientific). The qPCR analyses were performed on a 7500 Real-Time PCR System using SYBR™ Green (Thermo Fisher Scientific). Primers are outlined in Table 5. GAPDH was used as reference gene. The relative expression and fold change of each gene was calculated using the delta delta Ct method.

### RNA *in situ* hybridisation

The BaseScope™ (Advanced Cell Diagnostics, CA, USA) assays were performed manually according to the manufacturer’s instructions. This method allows the detection of exon junctions and the analysis of splice variants. Briefly, the BaseScope™ assay procedure included the following steps: FFPE sections were deparaffinised and treated sequentially with specific pre-treatments to allow for target probe access. Target probes were added onto the slides and incubated in the HybEZ oven (Advanced Cell Diagnostics) for 2 h at 40 °C to allow probe hybridization to RNA targets. The slides were washed and incubated with a series of signal amplification solutions. The signal was amplified using a multi-step process, and detected using a red chromogenic substrate (10 min at room temperature). The slides were counterstained with haematoxylin and mounted with Cytoseal mounting medium (Richard-Allan Scientific, CA, USA).

### GEO files

The data discussed in this publication have been deposited in NCBI’s Gene Expression Omnibus^28^ and are accessible through GEO Series accession number GSE118959 (https://www.ncbi.nlm.nih.gov/geo/query/acc.cgi?acc=GSE118959).

## Supplementary information

Dataset 1

Dataset 2

Dataset 3
